# Breakfast Skipping in a Multi-Ethnic Population of Middle-Aged Men and Relationship With Sociodemographic Variables and Weight Status

**DOI:** 10.3389/fnut.2021.761383

**Published:** 2022-02-02

**Authors:** Nora A. AlFaris, Naseem M. Alshwaiyat, Hana Alkhalidy, Reham I. Alagal, Jozaa Z. AlTamimi, Nora M. AlKehayez

**Affiliations:** ^1^Department of Physical Sports Sciences, College of Education, Princess Nourah bint Abdulrahman University, Riyadh, Saudi Arabia; ^2^School of Nutrition and Dietetics, Faculty of Health Sciences, Universiti Sultan Zainal Abidin, Terengganu, Malaysia; ^3^Department of Nutrition and Food Technology, Faculty of Agriculture, Jordan University of Science and Technology, Irbid, Jordan

**Keywords:** breakfast skipping, multi-ethnic, middle-aged men, weight status, body mass index

## Abstract

**Background:**

Breakfast eating is regarded to be necessary for maintaining a healthy body weight. On the other hand, breakfast skipping has been linked with obesity incidence. This study was carried out to determine the prevalence of breakfast skipping among a multi-ethnic group of middle-aged men living in Saudi Arabia and the association between breakfast skipping and sociodemographic variables and weight status.

**Methods:**

This cross-sectional study included 1,800 middle-aged men aged 36–59 years. Participants' sociodemographic information and frequency of breakfast eating were obtained through personal interviews. The body mass index was determined after measuring body weight and height using standardized methods.

**Results:**

The prevalence of breakfast skipping was 42.1% of the study participants. Nationality was a predictor of breakfast skipping. Bangladeshi participants (*N* = 100) have the lowest rate of breakfast skipping (9.0%), whilst Saudi participants (*N* = 161) have the highest rate (73.3%). Weight status was another predictor of breakfast skipping as breakfast skippers had a significantly higher average body mass index (27.1 ± 3.8) than breakfast consumers (26.2 ± 3.5). Overweight/obese participants have a significantly higher breakfast skipping rate (44.9%) than participants with underweight/normal weight (36.6%).

**Conclusion:**

The rate of breakfast skipping is relatively high among middle-aged men living in Saudi Arabia. The data support a link between breakfast skipping and sociodemographic variables and weight status.

## Introduction

Overweight and obesity prevalence is steadily increasing worldwide (1). Currently, obesity is the most frequent form of malnutrition, and it is associated with a higher incidence of obesity-related diseases and a higher global disease burden (2–4). Many dietary habits are thought to be linked to obesity, such as the number of meals consumed during the day, the number of meals consumed away from home, eating at night, the portion size of food, and skipping breakfast (5–8). Although the definition of breakfast is inconsistent in these studies, breakfast is typically characterized as the first meal of the day after waking up in the morning (9). Breakfast is an important meal since it supplies energy and essential nutrients to the body after a long time of fasting during the night (10). Breakfast consumption is connected to better food quality. Breakfast eaters consumed significantly more calcium and folate and consumed significantly less overall fat than breakfast skippers (11).

People's food habits have been shifted dramatically during the last few decades due to changes in their typical lifestyles such as high fast food consumption, sedentary behaviors, disturbs in sleep and wake up times and lack of time in the morning to prepare and eat breakfast. As a result, different population groups, including children, adolescents, and adults, are increasingly skipping breakfast (12, 13). Middle-aged adulthood is a stage of life marked by forming personal identity and stability in lifestyle and dietary behaviors. However, certain harmful eating habits, such as skipping breakfast, are still common among middle-aged adults (14). In recent years, breakfast skipping has become a controversial public health concern. Many research efforts studied the relationship between breakfast skipping and weight status. Nevertheless, the results of these studies are inconsistent. Breakfast skipping has been linked to a rise in the prevalence of obesity and obesity-related comorbidities in several studies (15–17). However, some studies found no connection between breakfast skipping and weight status, while others have claimed that breakfast skipping can help lose body weight (18, 19). Breakfast skipping has also been related to a higher risk of various non-communicable diseases, such as cardiovascular disease, type 2 diabetes, and certain malignancies (20–22).

Numerous mechanisms have been postulated to explain the relationship between skipping breakfast and obesity. Breakfast skipping has been associated with increased hunger and decreased satiety. This could result in overeating and impaired insulin sensitivity. On the other hand, breakfast eating can aid in controlling appetite while also improving insulin sensitivity for the next meal (16, 23). Furthermore, fasting from the previous night is interrupted by eating breakfast. The longer the fasting period, the higher the concentration of ghrelin, the hunger-inducing peptide hormone that can mimic fasting to boost human hedonic, orbitofrontal cortex, and hippocampal responses to eating (24). Obese adults who skip breakfast have a partial dietary compensation and consume more energy in the next meals throughout the day (25, 26). Moreover, skipping breakfast may increase the risk of obesity by affecting gene expression and hormone production. Skipping breakfast led to higher blood glucose levels after lunch and dinner, lower intact glucagon-like peptide-1 (iGLP-1) levels, and greater insulin resistance (27). Breakfast skipping affected the expression of genes involved in the circadian clock and metabolism, altering circadian hormone synthesis and elevating postprandial blood glucose levels (28). Breakfast skipping also causes stress-independent overactivity in the hypothalamic-pituitary-adrenal (HPA) axis, causing cortisol rhythm disruption (29).

Saudi Arabia is among the world's largest oil producers, with a rapidly expanding economy. Thus, Saudi Arabia attracts workers worldwide, particularly from the Middle East, South Asia, and Southeast Asia. Expatriates made up more than half of the total workforce in Saudi Arabia (30). Non-Saudi residents comprised about a third of the population, with three-quarters males (31). Migrants of various ethnic backgrounds offer a unique chance to investigate disparities in dietary behaviors and their links to health and disease in a varied community. Hence, the present study was conducted to determine the prevalence of breakfast skipping among a multi-ethnic sample of middle-aged men living in Saudi Arabia and the association between breakfast skipping and sociodemographic variables and weight status.

## Methods

### Study Design and Participants

This study is part of a research project entitled the Relationship between Obesity, physical Activity, and Dietary pattern among men in Saudi Arabia (ROAD-KSA). This research project is a cross-sectional study that aims to determine the prevalence of overweight and obesity, physical activity level, and dietary patterns among young and middle-aged men in Riyadh, Saudi Arabia, and the correlations between these factors. The present study was conducted in Riyadh, Saudi Arabia.

The recruitment of study participants was carried out randomly from public places in Riyadh city using a stratified clustered sampling technique according to geographic locations in the city. The study inclusion criteria were middle-aged men aged 36–59 years, living in Riyadh, free of any physical impairment, and have a single nationality of one of the following countries: Saudi Arabia, Egypt, Yemen, Syria, Jordan, Sudan, Turkey, Pakistan, Afghanistan, India, Bangladesh, and the Philippines. In accordance with the Helsinki Declaration, participants were asked to sign a consent form before taking part in the study. The research ethics committee of Princess Nourah bint Abdulrahman University in Riyadh, Saudi Arabia, approved the current study.

### Sociodemographic Variables

Sociodemographic variables data were collected by trained research assistants using personal interviews. The collected sociodemographic variables include the participants' nationality, age, residency period in Saudi Arabia, household type, marital status, educational level, and monthly income.

### Weight and Height Measurements

Participants' weight and height were measured by trained research assistants. A calibrated digital weight scale was used to measure the body weight to the nearest 0.1 kg while wearing little clothing and without shoes. Likewise, a calibrated portable stadiometer was used to measure the height to the nearest 0.1 cm while the body was in complete standing posture without shoes. The body mass index (BMI) calculation was done by dividing weight in kilogram by height in meter square. Study participants were divided to underweight (BMI < 18.5), normal (BMI = 18.5–24.9), overweight (BMI = 25–29.9), or obese (BMI ≥ 30) (32).

### Breakfast Consumption

A pre-tested questionnaire was used to assess breakfast eating frequency. An independent judgment from five experts in the field of nutrition research was used to assess the face validity of the questionnaire. To determine the reliability of our tool, a test-retest pilot study with a 2-week gap was conducted. Data from 60 men from the target population were acquired for a pilot study, but they were not included in the study sample. Personal interviews were used to obtain data by qualified research assistants. Breakfast consumption was measured by asking individuals how many days per week they typically ate breakfast over the preceding year. The responses ranged from no days per week to seven days per week. Breakfast is defined as any food or beverage consumed between the hours of 5:00 a.m. and 11:00 a.m. after waking up (33). Breakfast skipping is defined as skipping breakfast at least once a week, a criterion that has been employed in earlier research (34–38).

### Statistical Analysis

Data analysis was handled using IBM SPSS Statistics for Windows (version 26. Armonk, New York, United States, 2019). Categorical variables were analyzed using the Chi-squared test and presented as frequencies and percentages. Continuous variables were analyzed using independent samples *t*-test and presented as means and standard deviations. Univariate and multivariate logistic regression analyses were performed to detect the factors related to breakfast skipping odds ratios. All reported *P*-values were made based on two-tailed tests. Differences were considered statistically significant at *P*-values < 0.05.

## Results

The current study involved the participation of 1,800 respondents. Table 1 shows participants' sociodemographic variables and body weight status stratified by breakfast intake patterns. Our results revealed that 42.1% of the participants were breakfast skippers. By nationality, Bangladeshi participants have the lowest rate of breakfast skipping (9.0%), whilst Saudi participants have the highest rate (73.3%). Furthermore, breakfast skippers had a significantly lower average age (40.7 ± 3.9) than breakfast consumers (41.1 ± 3.8). Participants residing in Saudi Arabia for 5 years or less had a significantly higher breakfast skipping rate (53.9%) than those residing in Saudi Arabia for 6 years or more (39.4%). Participants living within family households had a significantly higher breakfast skipping rate (56.9%) than those living within non-family households (33.5%). Similarly, single participants had a significantly higher breakfast skipping rate (53.7%) than married participants (40.5%). Unexpectedly, highly educated participants (college degree or more) had a significantly higher breakfast skipping rate (58.6%) than less educated participants (high school or less, 33.5%). Participants with high monthly income (≥1,000 USD) had a significantly higher breakfast skipping rate (53.1%) than participants with lower monthly income (33.3%). Interestingly, breakfast skippers had a significantly higher average BMI (27.1 ± 3.8) than breakfast consumers (26.2 ± 3.5). Overweight/obese participants had a significantly higher breakfast skipping rate (44.9%) than participants with underweight/normal weight (36.6%).

**Table 1:** Sociodemographic variables and body weight status of study participants stratified according to breakfast consumption patterns.

**Variables**	**Total**	**Breakfast consumers (7 days/week)**	**Breakfast skippers (0–6 days/week)**	*P*-value^*^
**All participants**	*N* = 1,800	1,042 (57.9%)	758 (42.1%)	
Nationality				**0.001**
Saudi	*N* = 161	43 (26.7%)	118 (73.3%)	
Egyptian	*N* = 161	82 (50.9%)	79 (49.1%)	
Yemeni	*N* = 115	56 (48.7%)	59 (51.3%)	
Syrian	*N* = 157	79 (50.3%)	78 (49.7%)	
Jordanian	*N* = 170	50 (29.4%)	120 (70.6%)	
Sudanese	*N* = 174	122 (70.1%)	52 (29.9%)	
Turkish	*N* = 247	176 (71.3%)	71 (28.7%)	
Pakistani	*N* = 144	86 (59.7%)	58 (40.3%)	
Afghan	*N* = 147	101 (68.7%)	46 (31.3%)	
Indian	*N* = 153	121 (79.1%)	32 (20.9%)	
Bangladeshi	*N* = 100	91 (91.0%)	9 (9.0%)	
Filipino	*N* = 71	35 (49.3%)	36 (50.7%)	
Age (years)	*N* = 1,800	41.1 (3.8)	40.7 (3.9)	**0.001**
Residency period in Saudi Arabia				**0.036**
1**–**5 years	*N* = 332	153 (46.1%)	179 (53.9%)	
6 years or more	*N* = 1,468	889 (60.6%)	579 (39.4%)	
Household type				**0.001**
Non-family household	*N* = 1,136	756 (66.5%)	380 (33.5%)	
Family household	*N* = 664	286 (43.1%)	378 (56.9%)	
Marital status				**0.001**
Single	*N* = 214	99 (46.3%)	115 (53.7%)	
Married	*N* = 1,586	943 (59.5%)	643 (40.5%)	
Education level				**0.001**
High school or less	*N* = 1,182	786 (66.5%)	396 (33.5%)	
College degree or more	*N* = 618	256 (41.4%)	362 (58.6%)	
Monthly income				**0.001**
Low (<1,000 USD)	*N* = 998	666 (66.7%)	332 (33.3%)	
High (≥1,000 USD)	*N* = 802	376 (46.9%)	426 (53.1%)	
Body mass index (kg/m^2^)	*N* = 1,800	26.2 (3.5)	27.1 (3.8)	**0.001**
Body weight status				**0.001**
Underweight/normal weight	*N* = 601	381 (63.4%)	220 (36.6%)	
Overweight/obesity	*N* = 1,199	661 (55.1%)	538 (44.9%)	

* *Categorical variables were analyzed by using Chi-squared test and expressed as frequencies and percentages. Continuous variables were analyzed by using independent samples t-test and expressed as means and standard deviations. Differences were considered statistically significant at P-value < 0.05, and significant values were presented in Bold type*.

Table 2 shows the odds ratios for breakfast skipping among all participants based on sociodemographic variables and BMI. Compared with participants from Bangladesh (have lowest rate of breakfast skipping), participants from other countries had a significantly higher odds ratios for being breakfast skippers, including Saudi Arabia [adjusted odds ratio (OR) = 68.35, 95% CI = 21.77–214.58], Egypt (adjusted OR = 7.53, 95% CI = 3.42–16.59), Yemen (adjusted OR = 9.31, 95% CI = 4.09–21.22), Syria (adjusted OR = 8.81, 95% CI = 3.59–18.64), Jordan (adjusted OR = 18.07, 95% CI = 7.89–41.36), Sudan (adjusted OR = 4.30, 95% CI = 1.99–9.32), Turkey (adjusted OR = 3.65, 95% CI = 1.69–7.88), Pakistan (adjusted OR = 4.83, 95% CI = 2.19–10.67), Afghanistan (adjusted OR = 3.45, 95% CI = 1.57–7.59), India (adjusted OR = 2.96, 95% CI = 1.33–6.60), and the Philippines (adjusted OR = 9.42, 95% CI = 3.93–22.57). Furthermore, getting older was linked to lower odds ratios for skipping breakfast (adjusted OR = 0.97, 95% CI = 0.94–1.00). Participants who had lived in Saudi Arabia for 6 years or more had lower odds ratios for skipping breakfast than those who had been there for 5 years or less (adjusted OR = 0.61, 95% CI = 0.46–0.81). Married participants had significantly lower odds ratios for skipping breakfast (adjusted OR = 0.48, 95% CI = 0.34–0.68) than single respondents. Breakfast skipping was significantly more common among participants with a high monthly income (1,000 USD or more) than those with a low monthly income (adjusted OR = 1.34, 95% CI = 1.01–1.78). Finally, an increased BMI was linked to an increased odds ratio for breakfast skipping (adjusted OR = 1.04, 95% CI = 1.01–1.07). Additionally, respondents who lived in a family environment had significantly higher odds ratios for skipping breakfast than those who did not (unadjusted OR = 2.63, 95% CI = 2.16–3.20). Breakfast skipping was significantly more common among those with a college degree or above than those with a lower education level (unadjusted OR = 2.81, 95% CI = 2.30–3.43). Nevertheless, multivariate analysis did not confirm these correlations.

**Table 2:** Odds ratios for breakfast skipping among study participants for sociodemographic variables and body mass index.

**Variables**	Unadjusted odds ratio^*^	**95% CI**	***P*-value**	Adjusted odds ratio^**^	**95% CI**	***P*-value**
Nationality						
Bangladeshi	1.00			1.00		
Saudi	27.75	12.86–59.85	**0.001**	68.35	21.77–214.58	**0.001**
Egyptian	9.74	4.60–20.65	**0.001**	7.53	3.42–16.59	**0.001**
Yemeni	10.65	4.90–23.16	**0.001**	9.31	4.09–21.22	**0.001**
Syrian	9.98	4.70–21.20	**0.001**	8.18	3.59–18.64	**0.001**
Jordanian	24.27	11.35–51.90	**0.001**	18.07	7.89–41.36	**0.001**
Sudanese	4.31	2.02–9.20	**0.001**	4.30	1.99–9.32	**0.001**
Turkish	4.08	1.95–8.53	**0.001**	3.65	1.69–7.88	**0.001**
Pakistani	6.82	3.18–14.60	**0.001**	4.83	2.19–10.67	**0.001**
Afghan	4.61	2.14–9.93	**0.001**	3.45	1.57–7.59	**0.002**
Indian	2.67	1.22–5.88	**0.014**	2.96	1.33–6.60	**0.008**
Filipino	10.40	4.54–23.80	**0.001**	9.42	3.93–22.57	**0.001**
Age (years)	0.97	0.95–1.00	**0.036**	0.97	0.94–1.00	**0.030**
Residency period in Saudi Arabia						
1–5 years	1.00			1.00		
6 years or more	0.56	0.44–0.71	**0.001**	0.61	0.46–0.81	**0.001**
Household type						
Non-family household	1.00			1.00		
Family household	2.63	2.16–3.20	**0.001**	0.95	0.65–1.37	0.770
Marital status						
Single	1.00			1.00		
Married	0.59	0.44–0.78	**0.001**	0.48	0.34–0.68	**0.001**
Education level						
High school or less	1.00			1.00		
College degree or more	2.81	2.30–3.43	**0.001**	0.95	0.67–1.34	0.774
Monthly income						
Low (<1,000 USD)	1.00			1.00		
High (≥1,000 USD)	2.27	1.88–2.75	**0.001**	1.34	1.01–1.78	**0.042**
Body mass index (kg/m^2^)	1.07	1.04–1.10	**0.001**	1.04	1.01–1.07	**0.016**

* *Univariate logistic regression analysis was used to test differences between breakfast skippers vs. breakfast consumers (reference group). Differences were considered statistically significant at P-value < 0.05, and significant values were presented in Bold type*.

** *Multivariate logistic regression analysis was used to test differences between breakfast skippers vs. breakfast consumers (reference group) after adjusting for participants' sociodemographic variables and body mass index. Differences were considered statistically significant at P-value < 0.05, and significant values were presented in Bold type*.

## Discussion

In this study, breakfast skipping was explored in a multi-ethnic group of middle-aged men residing in Saudi Arabia from twelve Middle Eastern and Asian countries. According to our data, breakfast skippers made up nearly two-fifths of the participants (42.1%). Breakfast skipping among adults has been explored in a number of previous studies. According to a study from Saudi Arabia, 49.9% of male college students have breakfast every day, while 50.1% skip breakfast once weekly at minimum (39). A study from the United States reported that breakfast skippers accounted for 26.1 and 21.2% of men and women, respectively (40). According to a population-based survey conducted among Iranian University students, 47.8% of male students and 43.8% of female students skip breakfast three times each week at a minimum (41). Another study from Serbia reported that 77.8% of adults consume breakfast daily, and the remaining adults (22.2%) skip breakfast at least once weekly (8). According to a Swedish survey, 90% of adults aged 25–74 years eat breakfast regularly, with only 10% of them skipping breakfast (7). Surprisingly, breakfast skipping was only detected in 4.5 and 2.3% of Spanish men and women aged 25–64 years, respectively (6). Breakfast skipping was observed in 23.1% of adults aged 20–75 in Japan (26.7% of men and 17.1% of women) (42). Finally, a study from Taiwan found that breakfast skipping was reported in 49.7 and 50.3% of men and women aged 18–64 years, respectively (43).

According to our findings, the prevalence of breakfast skipping varied greatly among participants from different countries. This discovery is in line with the results of several earlier studies. According to a study that collected breakfast eating data from adults in eight European countries, breakfast skipping rates ranged dramatically between countries (35). Breakfast skipping rates were somewhat high in Greece (54%), Slovenia (49%), Hungary (43%), and Switzerland (34%), while they were comparatively low in Belgium (18%), Norway (16%), Spain (14%), and the Netherlands (12%) (35). Breakfast skipping differed by ethnicity in a study conducted in the United States. Breakfast skipping was reported among 23% of non-Hispanic whites, 31.8% of non-Hispanic blacks, and 19.5% of Hispanic American adults (40).

Based on the results of this study, breakfast skipping was also linked to age, household type, marital status, educational level, and monthly income. Our findings are in agreement with earlier research, which found that breakfast skippers are significantly younger than breakfast consumers (40–42), and married people have significantly a lower rate of breakfast skipping compared with single individuals (40, 41). Contrarily, our findings opposing findings from former studies reported that breakfast skipping was significantly higher among participants with low education compared with those with high education (35), and participants with low income compared with those with high income (44, 45).

Unsurprisingly, our findings confirmed the relationship between skipping breakfast and weight status. This relationship was verified among participants from different ages, genders, regions, and socioeconomic backgrounds. This point to a universal relationship between breakfast skipping and weight status (16). According to a meta-analysis of cross-sectional studies, breakfast skipping is linked to a 75% increase in obesity risk compared to those who have breakfast regularly (13). A recent systematic review and meta-analysis reported that breakfast skipping raised the risk of overweight and obesity by 48 and 44% based on data from cross-sectional and cohort studies, respectively (16). Breakfast skipping has been linked to obesity in several cohort studies. A 3-year retrospective cohort study of Japanese men (20–49 years) revealed that breakfast skipping was a predictor of being overweight (46). A prospective cohort study of American men aged 40–75 years revealed that breakfast consumption was inversely associated with the risk of five kilograms weight gain after 10 years of follow-up (47). A longitudinal population-based cohort from Sweden with a 27-year follow-up found that breakfast skipping in adolescence aged 16 years old predicted the metabolic syndrome and was associated with central obesity and high fasting glucose at middle-aged adulthood (43 years old) (14).

The current study has a few limitations. First, the cause-effect relationship cannot be determined because of the cross-sectional design for this study. Second, the present study did not account for the effect of daily energy consumption on BMI since no data on daily energy intake was available for analysis. Third, the scientific literature lacks a clear definition for breakfast skipping, resulting in bias. Nevertheless, this study used a large multi-ethnic population, and the findings of this study add to the growing body of knowledge that suggests the efficacy of breakfast eating in the prevention of obesity.

## Conclusions

In this study, breakfast skipping was relatively common among a multi-ethnic sample of middle-aged men in Saudi Arabia. Among middle-aged men from various nationalities living in Saudi Arabia, breakfast skipping rates varied significantly. Our data supported the link between breakfast skipping and sociodemographic variables and weight status. More studies applying robust designs, multi-ethnic samples, and various sex and age groups are needed to confirm the breakfast skipping relationship with sociodemographic variables and weight status.

## Data Availability Statement

The raw data supporting the conclusions of this article will be made available by the authors, without undue reservation.

## Ethics Statement

The studies involving human participants were reviewed and approved by the Research Ethics Committee at Princess Nourah bint Abdulrahman University, Riyadh, Saudi Arabia. The patients/participants provided their written informed consent to participate in this study.

## Author Contributions

JA and NAlF: conceptualization. NAlK and JA: methodology. JA, NAlK, RA, and NAlF: software. JA: validation, visualization, and project administration. NAlF, HA and NAls: formal analysis and data curation. RA: investigation and funding acquisition. NAlF: resources and supervision. JA and RA: writing—original draft preparation. NAls and HA: writing—review and editing. All authors have read and agreed to the published version of the manuscript.

## Funding

This research was supported by Princess Nourah bint Abdulrahman University Researchers Supporting Project number (PNURSP2022R34), Princess Nourah bint Abdulrahman University, Riyadh, Saudi Arabia.

## Conflict of Interest

The authors declare that the research was conducted in the absence of any commercial or financial relationships that could be construed as a potential conflict of interest.

## Publisher's Note

All claims expressed in this article are solely those of the authors and do not necessarily represent those of their affiliated organizations, or those of the publisher, the editors and the reviewers. Any product that may be evaluated in this article, or claim that may be made by its manufacturer, is not guaranteed or endorsed by the publisher.

## Abbreviations

BMI: body mass index
OR: odds ratio.
